# Evolution of the Onion Ring Sign in Radiation Retinopathy

**DOI:** 10.7759/cureus.97758

**Published:** 2025-11-25

**Authors:** Mustafa Kayabaşı, Ezgi Karataş, Ziya Ayhan, Ömer Kartı, Ali Osman Saatci

**Affiliations:** 1 Department of Ophthalmology, Muş State Hospital, Muş, TUR; 2 Department of Ophthalmology, Ağrı İbrahim Çeçen University, Ağrı, TUR; 3 Department of Ophthalmology, Dokuz Eylül University, İzmir, TUR

**Keywords:** hyperreflective crystalline deposits, intravitreal injection, onion ring sign, optical coherence tomography, radiation retinopathy

## Abstract

A 53-year-old male with a history of radiation therapy for thyroid ophthalmopathy was followed up for chronic bilateral vision loss and macular edema refractory to multiple intravitreal treatments for over 19 years. His clinical course included multiple intravitreal injections including triamcinolone acetonide, bevacizumab, pegaptanib, ranibizumab, aflibercept 2 and 8 mg, and dexamethasone implant. At the 17th year of follow-up, spectral-domain optical coherence tomography (OCT) revealed intensely hyperreflective, horizontally layered deposits within intraretinal cystic spaces without posterior shadowing, consistent with the onion ring sign. Fundoscopic examination showed highly refractile, golden-yellow lipid exudates corresponding to these OCT findings. Unlike previously reported cases of age-related macular degeneration and diabetic retinopathy, where these deposits were located primarily beneath or above the retinal pigment epithelium, the deposits in this patient were located within the inner retinal layers. The present case demonstrates that the onion ring sign may develop in long-standing radiation retinopathy, likely reflecting the cumulative effects of chronic vascular injury and persistent macular edema. The recognition of this OCT feature may aid in understanding the natural history, chronicity, and metabolic burden of retinal vascular disorders.

## Introduction

Cholesterol crystal formation is thought to be driven by chronic vascular inflammation, particularly in individuals with impaired lipid metabolism. These crystals typically result from the precipitation of lipids within lipid-laden macrophages and have been implicated in various systemic and ocular diseases [1]. Optical coherence tomography (OCT) has enabled the identification of a distinct linear and layered accumulation of cholesterol crystals in the subretinal pigment epithelium-basal laminar space, characterized by the absence of posterior shadowing [2,3]. These structures are described as hyperreflective crystalline deposits in eyes with non-neovascular age-related macular degeneration (AMD), and as the “onion sign” (or “onion ring sign”) in neovascular AMD, particularly in association with type 1 macular neovascularization [2,4]. More recently, this characteristic OCT finding has also been demonstrated in eyes with diabetic retinopathy, where the lesions are observed above the retinal pigment epithelium (RPE) [5].

Radiation retinopathy is a chronic, progressive, and vision-threatening complication resulting from exposure to various sources of ionizing radiation, typically developing after six months post-exposure [6]. Characteristic retinal findings include microaneurysms, capillary nonperfusion, telangiectasia, and retinal hemorrhages, which may lead to neovascularization and vision loss [6-8].

Here, we report the onion ring sign formation in a patient with long-standing treatment-resistant radiation retinopathy following an exceptionally long follow-up period of 19 years. To our best knowledge, this is the first report in the literature demonstrating the presence of onion ring sign in radiation retinopathy. It also highlights the importance of recognizing this distinctive feature, as it may provide insight into disease chronicity, reflect longstanding vascular compromise, and indicate treatment resistance.

## Case presentation

A 53-year-old male patient presented to our department in 2006 with the complaints of decreased vision in both eyes. He had no history of systemic disease and was not taking any medications. He had undergone radiotherapy for thyroid ophthalmopathy in 2003, with visual decline starting one year post-treatment. In 2005, the patient underwent vitrectomy in the left eye for central retinal vein occlusion at another institution.

**Figure 1:**
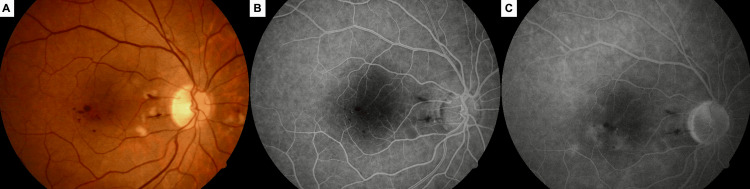
Right eye two weeks after the initial simultaneous intravitreal injection of triamcinolone acetonide and bevacizumab. Color fundus photograph (A) showing macular edema, hard exudates, and occasional punctate hemorrhages at the posterior pole. Early-phase (B) and late-phase (C) fluorescein angiograms revealing hyperfluorescence consistent with vascular leakage and hypofluorescent spots corresponding to retinal hemorrhages.

Best-corrected visual acuity was counting fingers at 4 meters in the right eye and at 2 meters in the left eye. Slit-lamp examination was unremarkable, and intraocular pressures were within normal limits in both eyes. Dilated fundus examination revealed bilateral macular edema, hard exudates, and occasional punctate hemorrhages at the posterior pole, along with a pale optic disc in the left eye. Fluorescein angiography and time-domain OCT (Stratus OCT; Carl Zeiss Meditec, Dublin, CA, United States) confirmed the presence of bilateral macular edema. Images from the initial clinical examination are not included, as they have been previously published in a separate case report [9]. Right intravitreal 2-mg triamcinolone acetonide (Kenacort-A®, Bristol-Myers Squibb) injection was administered in combination with 1.25-mg bevacizumab (Avastin®, F. Hoffmann-La Roche AG, Switzerland). Figure 1 shows the fundoscopic and fluorescein angiographic findings of the right eye two weeks after treatment.

**Figure 2:**
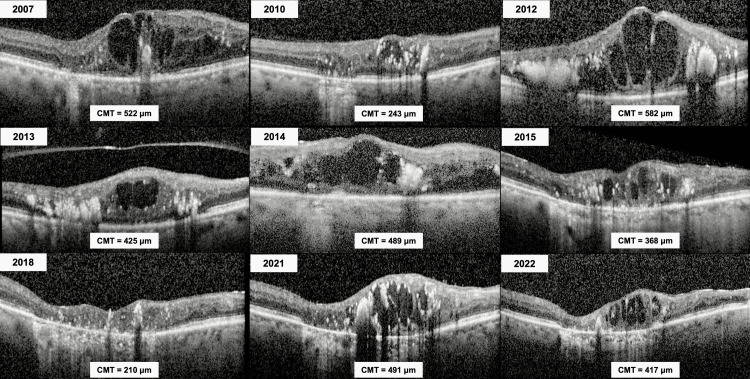
Longitudinal spectral-domain optical coherence tomography follow-up of the right eye from 2007 to 2022, illustrating the fluctuating course of macular edema and intraretinal exudation. The corresponding CMT measurements are provided for each time point. CMT, central macular thickness

The patient’s right eye received six combined intravitreal injections of triamcinolone acetonide and bevacizumab, one injection of 0.3 mg pegaptanib (Macugen®, Pfizer Inc., New York, NY, United States), four injections of 2 mg triamcinolone acetonide alone, 17 injections of 1.25 mg bevacizumab alone, 3 injections of 0.5 mg ranibizumab (Lucentis®, Novartis, Basel, Switzerland), 24 injections of 2 mg aflibercept (Eylea®, Bayer, Leverkusen, Germany), 16 dexamethasone intravitreal implant (0.7 mg, Ozurdex®, Allergan, Dublin, Ireland), and a single 8 mg aflibercept (Eylea HD®, Bayer, Leverkusen, Germany) during the 19-year follow-up. Additionally, focal laser photocoagulation and phacoemulsification combined with intraocular lens implantation were performed. The treatment history, including the number and type of intravitreal injections administered in the right eye over the 19-year follow-up, is summarized in Table 1. All treatments were administered exclusively to the right eye. No further treatment was administered to the left eye, as visual improvement was deemed unlikely due to pre-existing radiation-induced optic neuropathy, and the patient declined additional intervention based on the poor visual prognosis. Figure 2 shows the longitudinal spectral-domain optical OCT (Heidelberg Engineering, Heidelberg, Germany) follow-up until 2023, demonstrating the fluctuating course of macular edema and intraretinal exudation.

**Table 1:** Summary of treatments performed over 19 years of follow-up. VEGF, vascular endothelial growth factor

Treatment type	Agent/Procedure	Number of applications	Notes
Intravitreal corticosteroid	Triamcinolone (2 mg)	6 (combined with bevacizumab) 4 (alone)	Adjunct to anti-VEGF therapy; used in early treatment phase
Intravitreal anti-VEGFs	Pegaptanib (0.3 mg)	1	-
	Bevacizumab (1.25 mg)	17	Primary anti-VEGF agent during the initial years
	Ranibizumab (0.5 mg)	3	Partial anatomical response
	Aflibercept (2 mg)	24	Maintained as mainstay therapy from 2014–2023 along with dexamethasone implant
	Aflibercept (8 mg)	1	High-dose therapy introduced in Turkey in 2025
Steroid implant	Dexamethasone implant (0.7 mg)	16	Maintained as mainstay therapy from 2014 to 2023 along with aflibercept
Laser treatment	Focal/grid laser photocoagulation	-	Applied to areas of focal leakage and ischemia in the right eye
Surgical treatment	Phacoemulsification with intraocular lens implantation	-	2018

**Figure 3:**
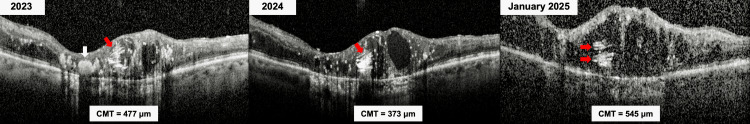
Transfoveal spectral-domain optical coherence tomography sections of the right eye following detection of the onion ring sign (red arrows), demonstrating intensely hyperreflective deposits within intraretinal cystic spaces arranged in a linear and horizontal pattern. Notably, these deposits lack posterior shadowing, in contrast to hard exudates (white arrow). The corresponding CMT measurements are provided for each time point. CMT, central macular thickness

**Figure 4:**
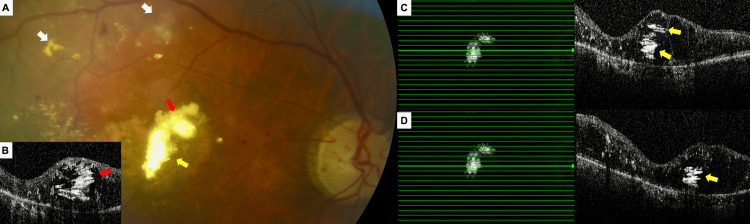
Right eye at the last visit. Color fundus photograph (A) showing highly refractile, golden-yellow lipid exudates at the fovea (red and yellow arrows), perifoveal chorioretinal atrophy, and pale optic disc, along with hard exudates (white arrows) and occasional retinal hemorrhages in the superior-temporal macular region. Consecutive spectral-domain optical coherence tomography scans (B–D) demonstrating the onion ring sign (red and yellow arrows) corresponding to the highly refractile golden-yellow exudation areas observed on the color fundus photograph. Central macular thickness measured 594 μm.

Since 2023, we noticed the formation of the onion ring sign and its evolution on the consecutive OCT sections of the right eye, characterized by intensely hyperreflective deposits within intraretinal cystic spaces, arranged in a linear and horizontal pattern and lacking posterior shadowing (Figure 3). A systemic lipid evaluation was performed at this stage to assess possible systemic dyslipidemia, which resulted in normal cholesterol and triglyceride levels. At the last visit in July 2025, the patient’s best-corrected visual acuity was counting fingers at 3 meters in the right eye and at 1 meter in the left eye. Fundoscopic examination revealed highly refractile, golden-yellow lipid exudates at the fovea, perifoveal chorioretinal atrophy related to previous laser photocoagulation along with hard exudates, and occasional retinal hemorrhages in the superior temporal macular region (Figure 4A). Consecutive spectral-domain OCT scans in years revealed intensely hyperreflective horizontal deposits without posterior shadowing within the intraretinal cystic spaces consistent with the onion ring sign, corresponding to the highly refractile, golden-yellow exudation areas observed on fundoscopic examination (Figures 4B-4D).

**Figure 5:**
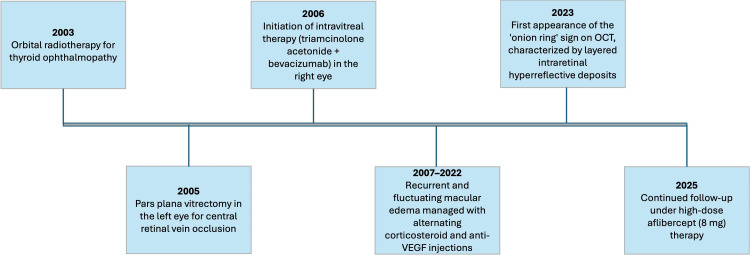
Summary timeline depicting key imaging milestones, detection of the onion ring sign, and major treatment events during the 19-year follow-up. VEGF, vascular endothelial growth factor

A summary timeline integrating clinical and imaging milestones is presented in Figure 5.

## Discussion

The present case highlights the formation and evolution of the onion ring sign in a patient with radiation retinopathy over a long-term follow-up period, despite frequent and intensive intravitreal therapy, including multiple administrations of dexamethasone intravitreal implants and even high-dose aflibercept. To the best of our knowledge, this is the first case of the onion ring sign in the context of radiation-induced macular edema, and it represents one of the longest followed up radiation retinopathy case in the literature.

In 2021, Mukkamala et al. [10] first described the onion ring sign as a novel spectral-domain OCT finding characterized by layered hyperreflective lines within the sub-RPE space, typically associated with chronic exudation from type 1 choroidal neovascularization in AMD. The authors speculated that these stratified reflective lesions represented precipitated lipid, collagen, or fibrin, resulting from longstanding exudative activity [10]. This hypothesis was later substantiated by a histopathological study conducted by Pang et al. [4], who provided histologic evidence that ex vivo cholesterol clefts corresponded to the in vivo OCT manifestation of the onion ring sign, thereby supporting the notion that these hyperreflective lamellar structures represent lipid-rich material accumulated over time in chronically exudative retinal conditions.

Although the onion ring sign was initially described in eyes with neovascular AMD [2,4,11], Venkatesh et al. [5] recently reported similar OCT findings in eyes with diabetic retinopathy. Notably, these lesions were localized within the outer retinal layers, situated above the RPE, in contrast to their typical sub-RPE localization observed in AMD. Unlike hard exudates, these lesions did not exhibit posterior shadowing on spectral-domain OCT. The authors postulated that these findings represent cholesterol crystal deposits similar to those observed in AMD. Furthermore, they suggested that the presence of the onion ring sign in diabetic maculopathy might indicate chronic vascular leakage and underlying hypercholesterolemia, potentially requiring lipid-lowering therapy. These deposits may be associated with significant visual impairment when the fovea is involved. The presence of the onion ring sign involving the fovea, along with the impaired visual acuity, as observed in the present case, further supports these conclusions. Notably, our patient had no systemic diseases, including hypercholesterolemia, and was not receiving any medications, including lipid-lowering therapy.

Radiation retinopathy shares several pathological mechanisms with diabetic retinopathy, such as capillary nonperfusion, endothelial cell damage, and chronic disruption of the blood-retinal barrier, all of which may contribute to the gradual accumulation of lipids and their eventual crystallization [6,8]. In the literature, radiation retinopathy cases have predominantly been reported following radiotherapy for various intraocular, cranial, or head and neck malignancies such as retinoblastoma, choroidal malignant melanoma, or nasopharyngeal carcinoma [12,13]. Due to the often-fatal nature of these primary diseases, long-term follow-up of affected patients was typically limited. In contrast, the present case involved radiotherapy for a non-malignant, non-fatal condition - thyroid ophthalmopathy - allowing for extended longitudinal follow-up. Despite intensive multimodal intravitreal therapy, unresolved macular edema created a permissive environment for cholesterol crystal formation. The delayed onset of the onion ring sign, emerging more than 17 years after the initial diagnosis, suggests that chronicity and lipid burden, rather than acute inflammation, are the critical drivers of crystal deposition. Interestingly, unlike diabetic retinopathy, in which deposits localize to the outer retinal layers just above the RPE, this case demonstrated the onion ring sign within the intraretinal cystic spaces, suggesting that disease-specific fluid dynamics influence the pattern of cholesterol crystal deposition.

Recognition of the onion ring sign in radiation retinopathy may have significant clinical implications for patient management, as its presence could indicate progression to a chronic, treatment-resistant phase of the disease. Identifying this particular sign may assist clinicians in optimizing treatment intervals and follow-up schedules, as well as in counseling patients regarding their long-term visual prognosis. The onion ring sign may be mistaken for hard exudates due to their similar appearance. However, hard exudates typically present as discrete, dome-shaped or round hyperreflective lesions with prominent posterior shadowing on OCT, whereas the onion ring sign is characterized by lamellar, linearly arranged hyperreflective bands without any associated shadowing. Recognizing this distinction is essential to avoid misinterpretation in cases of chronic vascular leakage.

## Conclusions

The present case expands the clinical spectrum of the onion ring sign by demonstrating its occurrence in radiation retinopathy at the 17th year of follow-up. The delayed onset of this finding underscores the role of chronicity and long-term lipid accumulation, rather than acute inflammation, in cholesterol crystal deposition. Awareness of this OCT feature may provide valuable insights into disease chronicity and guide future studies on its prognostic significance and potential therapeutic implications.
